# Automated segmentation and clinical characterization of layered plaque in intracoronary optical coherence tomography

**DOI:** 10.1016/j.ijcha.2026.102025

**Published:** 2026-10-09

**Authors:** Ruben G.A. van der Waerden, Joske L. van der Zande, Simone Saitta, Rick H.J.A. Volleberg, Jan-Quinten Mol, Pierandrea Cancian, Thijs J. Luttikholt, Xiaojin Gu, Leah Heil, Maarten van Leeuwen, Martijn Meuwissen, Alexey V. Protopopov, Peep Laanmets, Oleg V. Krestyaninov, Robert Dennert, Rohit M. Oemrawsingh, Jan-Peter van Kuijk, Karin Arkenbout, Dirk J. van der Heijden, Elvin Kedhi, Tomasz Roleder, Clara I. Sánchez, Bram van Ginneken, Ivana Išgum, Jos Thannhauser, Niels van Royen

**Affiliations:** a Department of Cardiology, Radboud University Medical Center, Nijmegen, the Netherlands; b Diagnostic Image Analysis Group, Radboud University Medical Center, Nijmegen, the Netherlands; c Department of Biomedical Engineering and Physics, Amsterdam University Medical Center, Amsterdam, the Netherlands; d Informatics Institute, University of Amsterdam, Amsterdam, the Netherlands; e Amsterdam Cardiovascular Sciences, Amsterdam UMC, the Netherlands; f Department of Cardiology, Isala Hospital, Zwolle, the Netherlands; g Department of Cardiology, Amphia Hospital Breda, the Netherlands; h Cardiovascular Center of Regional State Hospital, Krasnoyarsk, Russia; i Krasnoyarsk State Medical University, Krasnoyarsk, Russia; j Cardiology Center, North Estonia Medical Center, Tallinn, Estonia; k Meshalkin National Medical Research Center, Novosibirsk, Russia; l Department of Cardiology, Dr. Horacio E. Oduber Hospital, Oranjestad, Aruba; m Department of Cardiology, Albert Schweitzer Hospital, Dordrecht, the Netherlands; n Department of Cardiology, Sint Antonius Hospital, Nieuwegein, the Netherlands; o Department of Cardiology, Tergooi Hospital, Blaricum, the Netherlands.; p Department of Cardiology, Canisius Wilhelmina Hospital, Nijmegen, the Netherlands; q Department of Cardiology, Haaglanden Medical Center, The Hague, the Netherlands; r Department of Cardiology, McGill University Health Center, Royal Victoria Hospital, Montreal, Canada; s Faculty of Medicine, Wrocław University of Science and Technology, Wrocław, Poland; t Department of Radiology and Nuclear Medicine, Amsterdam University Medical Center, University of Amsterdam, Amsterdam, the Netherlands; u Department of Radiology, Mayo Clinic, Rochester, MN, USA

**Keywords:** Optical coherence tomography, Artificial intelligence, Layered plaque, Vulnerable plaque, Segmentation

## Abstract

**Background:**

Layered plaque, representing organized thrombus overlying a previously disrupted plaque, is a marker of coronary destabilization and plaque vulnerability. Despite its clinical relevance, identifying layered plaque remains expert dependent with interobserver variability, limiting large-scale assessment. We developed and validated an artificial intelligence (AI) model for layered plaque segmentation, and assessed occurrence and associations with plaque vulnerability in non-culprit lesions.

**Methods:**

Optical coherence tomography (OCT) pullbacks from the PECTUS-obs and ORANGE datasets were used to develop an enhanced version of our validated multiclass segmentation model (OCT-AID), by adding layered plaque as an additional pixel-level class. Pullbacks were divided into a training set (*n* = 4121 frames, 493 with layered plaque) and an independent held-out test set (*n* = 433 frames, 71 with layered plaque). Using this model, prevalence of layered plaque and associations with plaque vulnerability features were studied in patients with non-culprit lesions (PECTUS-obs data; 414 patients, 488 lesions).

**Results:**

The model achieved a Dice coefficient of 0.68 ± 0.27 for layered plaque segmentation, with pullback-level sensitivity of 96.2% and specificity of 75.0%. At least one layered plaque was present in 366/414 patients (88.4%). Presence of layered plaque was higher in STEMI patients (*p* *=* *0.018)*. Lesions containing layered plaques showed higher prevalence of lipid-rich plaque (76.4% vs. 60.9%, *p* *=* *0.008*), thin-cap fibroatheroma (29.2% vs. 15.6%, *p* *=* *0.016*), macrophage accumulation (25.0% vs 9.4%, *p* *=* *0.010*), and cholesterol clefts (0.0% vs. 8.3%; *p* *=* *0.009*).

**Conclusions:**

Automated OCT-based identification of layered plaque using AI is feasible. In non-culprit coronary lesions, layered plaque was highly prevalent and associated with established markers of plaque vulnerability.

## Graphical abstract

*AI, artificial intelligence; NSTEMI, non-ST-segment elevation myocardial infarction; OCT, optical coherence tomography; STEMI, ST-segment elevation myocardial infarction; TCFA, thin-cap fibroatheroma.*

**Figure:**
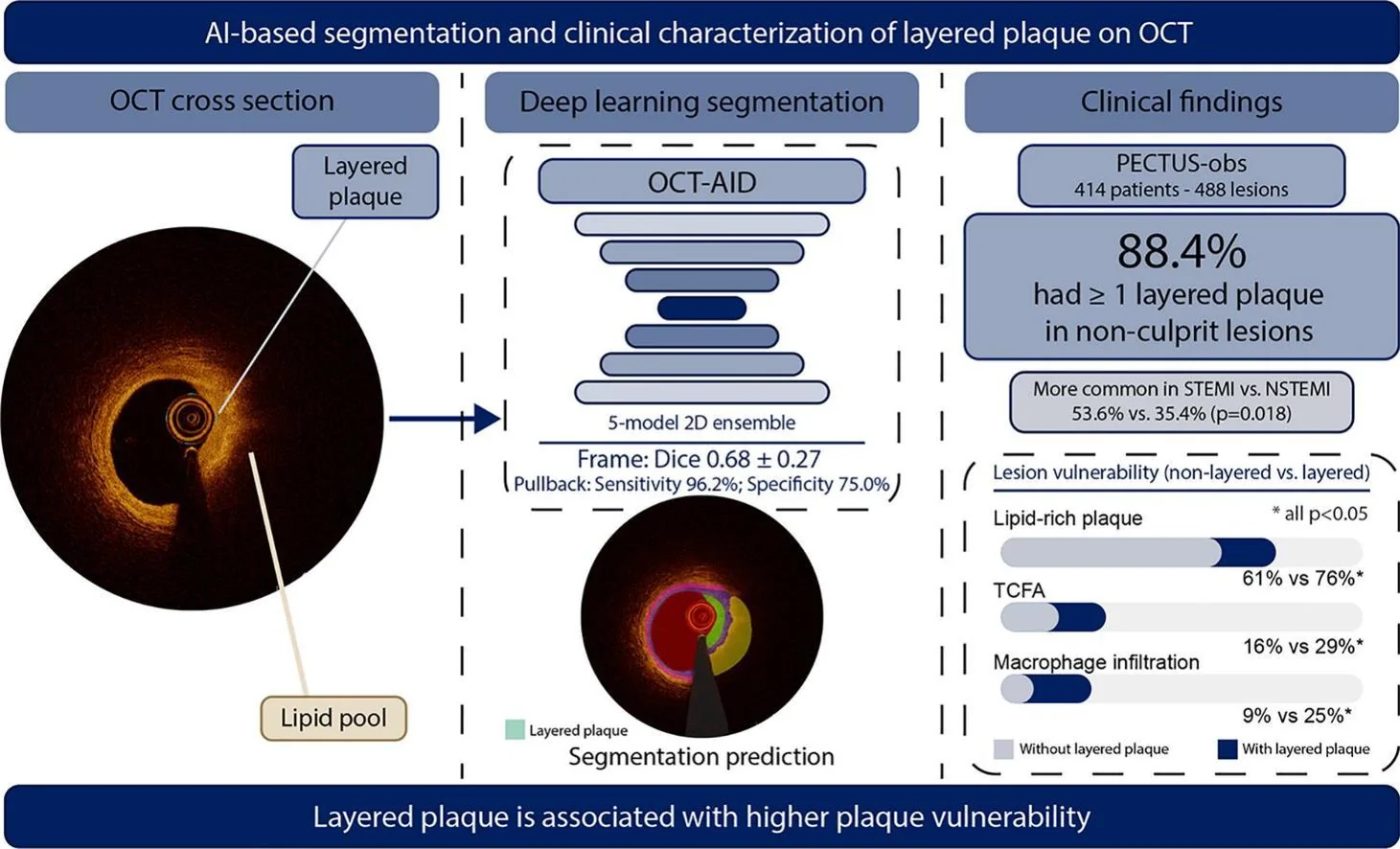

## 1 Introduction

Acute coronary syndrome (ACS) is caused by abrupt alterations in atherosclerotic plaque architecture, typically plaque rupture or erosion, leading to thrombus formation and myocardial injury. [1] While clinically apparent events define ACS presentation, many plaque ruptures occur silently. [2] Such subclinical episodes initiate a healing response characterized by thrombus organization and layered collagen deposition, resulting in a layered plaque morphology that reflects episodic plaque destabilization and contributes to rapid plaque progression. [3], [4] Autopsy studies demonstrated that layered plaques are highly prevalent in patients with sudden coronary or ischemic death, with reported prevalences of 61% and 73%, respectively. [5], [6]

Intracoronary optical coherence tomography (OCT) enables in vivo visualization of layered plaque, revealing signal-rich stratified patterns consistent with sequential thrombus organization and collagen maturation. [7] Histopathological evidence validated the ability of OCT to identify these layers and linked them to prior plaque disruption and healing. [8] However, OCT-based assessment of layered plaque remains challenging. Most prior work relied on manual, frame-level interpretation, limiting large scale assessment. Consequently, available data are largely descriptive and focused on culprit lesions [9], leaving the complete picture of layered plaque prevalence, distribution, and clinical relevance in non-culprit lesions, important contributors of recurrent ACS, undefined. [10]

Artificial intelligence (AI), particularly deep learning-based image segmentation, offers a potential solution to these limitations. Recent work has demonstrated the feasibility of automated layered plaque detection, although prior approaches were limited to frame-level classification [11] or ex vivo segmentation without in vivo evaluation. [12] Consequently, no current method provides robust, pixel-level segmentation of layered plaque within a unified multiclass framework suitable for morphological quantification.

In this study, we aimed to expand our previously validated OCT-based AI-segmentation model (OCT-AID) by incorporating layered plaque as a dedicated pixel-level class. First, we trained and evaluated the performance of this enhanced model on layered plaque. Second, we described layered plaque occurrence in non-culprit lesions of patients with recent myocardial infarction (MI) and assessed the association between layered plaque presence and established vulnerability features.

## 2 Methods

### 2.1 Study design

Data was used from the PECTUS-obs study (NCT03857971) and the ORANGE (Optical coherence tomography: pRognostic value and Artificial iNtelliGencE) registry (NCT07293858). All participants provided written informed consent.

The prospective, multicenter PECTUS-obs study investigated whether OCT-defined high-risk plaque features in fractional flow reserve (FFR-) negative non-culprit lesions are associated with subsequent major adverse cardiovascular events (MACE) in patients presenting with recent myocardial infarction. The full study protocol and primary outcomes have been reported previously. [13], [14] In brief, following successful treatment of the infarct-related artery, OCT was performed of the non-culprit lesion if invasive coronary angiography demonstrated at least one non-culprit lesion of intermediate severity (visually estimated diameter stenosis 30–90%) in any coronary vessel that was not hemodynamically significant (FFR > 0.80). The study adhered to the principles of the Declaration of Helsinki, and approval was obtained from the institutional review board or medical ethics committee at each participating center. For the present analysis, patients were included if at least one OCT pullback was of sufficient quality for evaluation.

The ORANGE registry is a prospective database of patients who underwent clinically indicated OCT imaging (Radboud University Medical Center, Nijmegen, the Netherlands). For the present study, a subset of pre-percutaneous coronary intervention (PCI) OCT pullbacks acquired between January 2021 and March 2025 was included. Pullbacks with sufficient image quality and mixed plaque morphologies were selected for analysis.

All OCT pullbacks were acquired using the Dragonfly Optis or Opstar imaging catheter (Abbott Vascular, USA) and exported for offline analysis. Acquisition, preprocessing, and frame-level handling followed previously published protocols. [15]

### 2.2 Dataset for model development

The reference standard for model training and evaluation was based on pixel-wise annotations generated by trained analysts with 2–5 years of OCT interpretation experience and supervised by an interventional cardiologist with >10 years of experience. Layered plaque was added as a new class in the existing annotated dataset and defined according to established OCT criteria: a plaque containing one or more discrete, signal-rich layers with clear demarcation from the underlying tissue. [16] Frames were systematically annotated at predefined intervals (one every 40 frames), to ensure representative coverage. In addition, intermediate frames were annotated when layered plaque morphology or other characteristic features were visually identified. Frames for model development were included only when complete pixel-wise annotation of all classes present in that frame was available. The final label set comprised the following classes: guidewire artifact, lumen, side branch, intima, media, lipid plaque, calcium plaque, layered plaque, thrombus, plaque rupture, neovascularization and background.

OCT data from both the PECTUS-obs and the ORANGE cohorts were used for model development. Datasets were combined and split at the patient level into training (90%) and held-out test (10%) sets, stratified by demographics and class presence. A 5-fold cross-validation strategy was applied within the training dataset to obtain robust estimates of segmentation performance, resulting in five independently trained models. These models were combined into an ensemble for inference. The held-out test set was used exclusively for final model evaluation.

Pullback-level sensitivity and specificity were assessed independently from the segmentation task. Two independent expert readers reviewed each pullback in the held-out test set and annotated the presence or absence of layered plaque at pullback level, providing the reference standard for this evaluation.

### 2.3 Dataset for clinical descriptive analysis

Clinical descriptive analyses were performed exclusively on the PECTUS-obs dataset. In this study, coronary lesions were identified and defined by an independent core laboratory based on coronary angiography. For each lesion, the core lab assessed plaque vulnerability features, including lipid-rich plaque, thin-cap fibroatheroma (TCFA), macrophage accumulation, thrombus, neovascularization, cholesterol clefts, and plaque rupture.

OCT pullbacks corresponding to these predefined lesions were used to describe the presence, volume, and morphology of layered plaque. Layered plaque identification for clinical analysis was based entirely on automated AI-generated segmentation outputs without manual correction. Only frames located within the core lab defined lesion were included.

### 2.4 Artificial intelligence for layered plaque segmentation

Layered plaque segmentation was implemented by extending our previously validated OCT-AID framework, [15] which performs multiclass pixel-wise segmentation of intracoronary OCT images, to include layered plaque as an additional output class. The model was developed based on the latest implementation of nnU-Net, [17], [18] a state-of-the-art framework for medical image segmentation. To incorporate spatial continuity and approximate expert visual assessment across sequential frames, the model was trained using a pseudo-3D input strategy. Specifically, seven consecutive cross-sectional OCT frames were stacked along the channel dimension, and the model generated a two-dimensional semantic segmentation for the central frame. Input images were grayscale, and a circular mask was applied to remove the manufacturer watermark prior to processing. Data augmentation strategies, including geometric (rotation, scaling, mirroring), photometric (brightness and contrast), and noise-based transformations (Gaussian noise, blur, and low-resolution), were implemented according to the default nnU-Net pipeline and consistent with the original OCT-AID development. [15] Training details on hyperparameters are presented in Supplemental Table 1. A detailed description regarding training procedures and ablation experiments is available in the original publication. [15]

During inference, connected-component analysis was applied as post-processing to remove small, isolated predictions for all classes except plaque rupture and thrombus, which were retained due to their naturally focal morphology. Frame-level performance for each class was assessed using sensitivity and specificity (95% confidence interval [CI]) for identification of class presence within a frame, calculated across the held-out test set. Segmentation performance was evaluated at the pixel-level using Dice similarity coefficient, calculated on frames containing ground-truth annotations for the respective class. Frame-level sensitivity and specificity (95% CI) of the AI model for identification of layered plaque presence were assessed against strict reader consensus using all frames from the pullbacks in the held-out test set, excluding frames classified as artefacted by either reader. Agreement between the AI model and strict reader consensus was assessed using Cohen's kappa. Inter-reader agreement was assessed using Cohen's kappa on the same non-artefacted frames. Pullback-level sensitivity and specificity (95% CI) of the AI model for identification of layered plaque presence were assessed against strict reader consensus using the held-out test set.

### 2.5 Clinical layered plaque characterization

Layered plaque was analyzed in a PECTUS-obs cohort using the framework previously established in our PECTUS-AI study, [19] where 414 patients comprising 488 lesions from the same dataset were evaluated.

At the frame-level, layered plaque segmentations were transformed from catheter-centered to lumen-centered coordinates to ensure consistent spatial alignment across frames and enable accurate volumetric reconstruction. Frames with severe attenuation artifacts, primarily due to residual blood, were identified using a previously validated artifact detection model. [20] A frame was excluded when >25% of its A-lines were classified as artifact-affected. Lesions were excluded when >50% of their frames met this exclusion criterion.

At the pullback-level, volumetric continuity of layered plaque segmentation was maintained by correcting single interruptions within otherwise continuous layered plaque segments. Single frame gaps, whether due to false-negative predictions or a single artifact-excluded frame, were filled by reclassifying only those pixels overlapping with layered plaque regions in both neighboring frames. Sequences of two or more consecutive artifact-affected frames were excluded from quantitative analysis.

After correction, layered plaque predictions were grouped based on three-dimensional spatial connectivity, with non-contiguous regions classified as separate layered plaques. Plaques present in only a single cross-sectional frame were reclassified as non-layered plaque and treated as noise. For each remaining plaque, volume (mm^3^), length (mm), maximum angle (°) and maximum thickness (μm; maximum distance between the adluminal and abluminal border of layered plaque) were derived from the reconstructed three-dimensional volume.

At the lesion level, total layered plaque volume (mm^3^) was calculated as the sum of all plaques, and the plaque with the largest volume was used to represent lesion-level morphology (length, angle, and thickness).

### 2.6 Statistical analysis

Categorical variables were summarized as counts (percentages). Continuous variables were expressed as means ± standard deviations (SD) when normally distributed or as medians with interquartile range (IQR) when non-normally distributed. Baseline characteristics and layered plaque measurements were described using standard descriptive statistics. Group comparisons were performed using the independent samples *t*-test for continuous variables and the chi-square test for categorical variables.

For patient-level analyses, patients were classified according to layered plaque presence such that, when multiple lesions were present, the patient was assigned to the layered-plaque group if any lesion contained layered plaque. Comparisons at the patient level were performed using chi-squared tests.

Lesion-level layered plaque metrics and structure variables were analyzed using generalized estimating equations (GEE) with an exchangeable correlation structure to account for within-patient clustering. Categorical outcomes were modeled using a logit link, and continuous outcomes were modeled using an identity link, except for variables following a gamma distribution, which were analyzed using a log link. For 2-group comparisons of vulnerability features, multivariable GEE analyses were additionally performed adjusting for clinical characteristics that differed significantly between groups. Where complete separation prevented GEE estimation, Fisher's exact test was used for 2-group comparisons and a Monte Carlo Fisher–Freeman–Halton exact test for 3-group comparisons.

All analyses were performed using IBM SPSS Statistics version 27.0.1.0 (IBM Corp., USA). A two-sided *p*-value <0.05 was considered statistically significant.

## 3 Results

### 3.1 Model development dataset characteristics

For the development and internal evaluation of the segmentation model, a total of 4554 images (267 patients, 300 pullbacks) were annotated at the pixel-level. Of these, 4121 images (241 patients, 270 pullbacks) were allocated to the training set and 433 images (26 patients, 30 pullbacks) to the held-out test set. In the training set, 493 images (12.0%) contained layered plaque, compared with 71 images (16.4%) in the held-out test set. The distribution of dataset origin was comparable between splits, with ORANGE and PECTUS-obs comprising 14.5% and 85.5% of the training set, and 15.4% and 84.6% of the test set, respectively (*p* = 0.906). The study flow of patients from the ORANGE registry and the PECTUS-obs study for model development is shown in Fig. 1. Baseline characteristics of the model development cohort are presented in Supplemental Table 2.

**Fig. 1:**
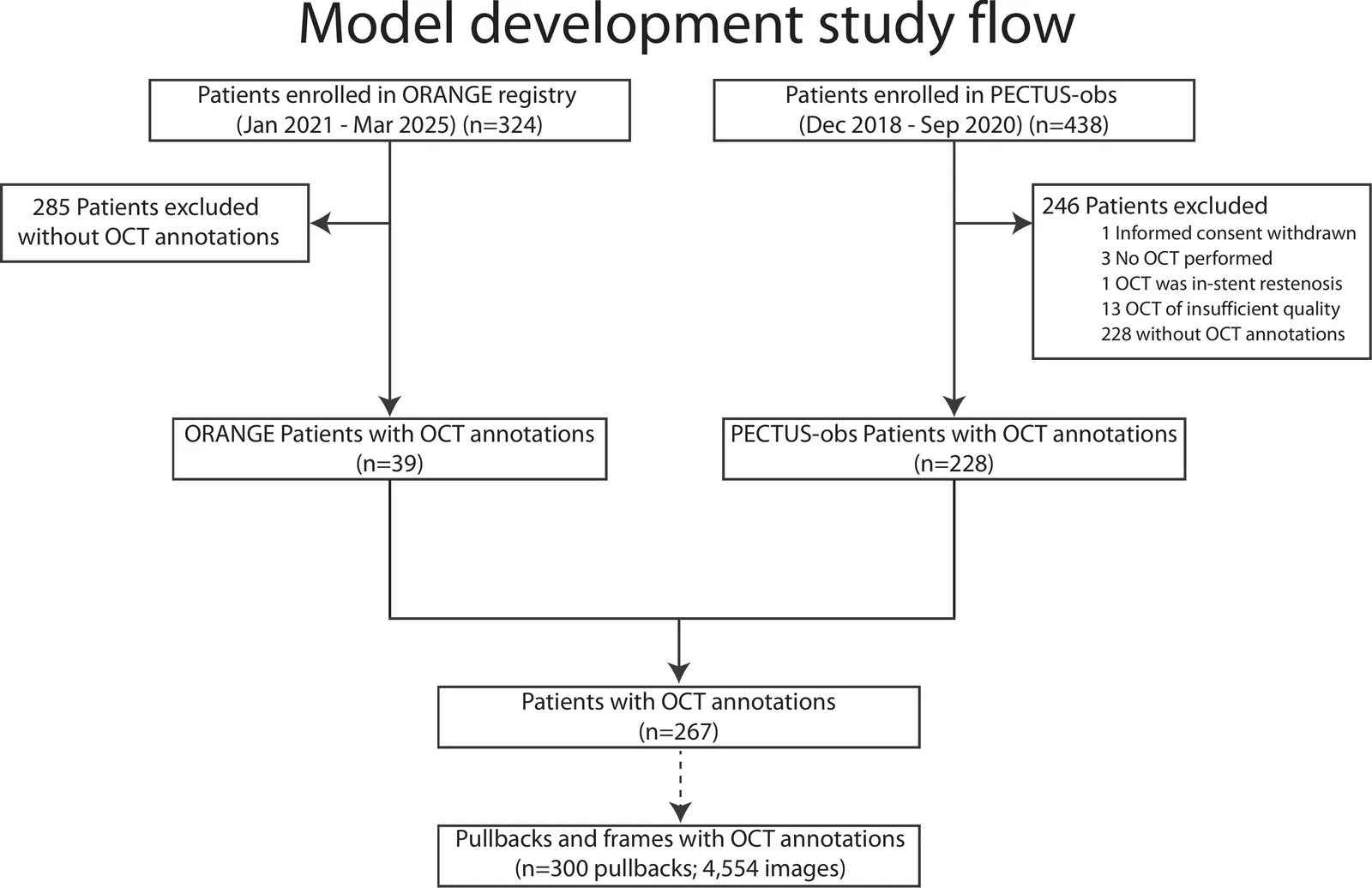
Study flow for model development. Flowchart of patient selection for segmentation model development using data from the ORANGE registry and the PECTUS-obs study. OCT, optical coherence tomography.

### 3.2 Model performance on layered plaques

Performance of all segmentation classes using the five-model 2D ensemble on the held-out test set is presented in Table 1. For layered plaque, pixel-level performance on frames containing ground-truth layered plaque yielded a Dice similarity coefficient of 0.68 ± 0.27. At the frame-level, sensitivity for identification of layered plaque presence was 56.3% (95% CI: 44.8–67.3), with a specificity of 96.1% (95% CI: 93.6–97.7).

**Table 1:** Segmentation performance metrics.

	Frame-level distribution (*n* = 4554)		Frame-level segmentation	Frame-level classification	
	Training set (*n* = 4121)	Held-out test set (*n* = 433)	Dice coefficient (−)	Sensitivity (%; 95% CI)	Specificity (%; 95% CI)
Layered plaque	493 (12.0%)	71 (16.4%)	0.68 ± 0.27	56.3% (44.8–67.3)	96.1 (93.6–97.7)
**Other classes**					
Guidewire artifact	4121 (100%)	433 (100%)	0.96 ± 0.04	100% (99.1–100)	100% (99.1–100)
Lumen	4121 (100%)	433 (100%)	0.99 ± 0.01	100% (99.1–100)	100% (99.1–100)
Sidebranch	809 (19.6%)	92 (21.3%)	0.84 ± 0.20	94.6% (87.9–97.7)	93.0% (89.7–95.2)
Intima	4121 (100%)	433 (100%)	0.89 ± 0.08	100% (99.1–100)	100% (99.1–100)
Media	3898 (94.6%)	424 (97.9%)	0.85 ± 0.11	99.3% (97.9–99.8)	77.8% (45.3–93.7)
Lipid plaque	2033 (49.3%)	218 (50.4%)	0.78 ± 0.16	94.5% (90.6–96.8)	81.9% (76.2–86.4)
Calcium plaque	1455 (35.3%)	170 (39.3%)	0.83 ± 0.20	94.7% (90.2–97.2)	93.5% (89.9–95.9)
Thrombus	435 (10.6%)	22 (5.1%)	0.81 ± 0.24	63.6% (43.0–80.3)	98.8% (97.2–99.5)
Plaque rupture	241 (5.9%)	23 (5.3%)	0.75 ± 0.26	73.9% (53.5–87.5)	97.1% (95.0–98.3)
Neovascularization	537 (13.0%)	53 (12.2%)	0.65 ± 0.26	60.4% (46.9–72.4)	95.8% (93.3–97.4)

Overview of frame-level dataset composition (training and held-out test sets) and OCT-AID segmentation performance for all classes, reported as pixel-level Dice coefficients and frame-level sensitivity and specificity with 95% confidence intervals.

*CI, Confidence interval.*

At the frame level, 10,483 valid frames from pullbacks in the held-out test set were evaluated for layered plaque presence against a strict consensus between two expert readers. Inter-reader agreement was substantial (κ = 0.79, 95% CI: 0.78–0.81). The AI model showed moderate agreement with expert consensus (κ = 0.52, 95% CI: 0.50–0.55), with a sensitivity of 47.8% (95% CI: 45.3–50.2%) and a specificity of 97.0% (95% CI: 96.6–97.3%) for identification of layered plaque presence.

At the pullback level, 30 held-out test set pullbacks were evaluated using the consensus of two expert readers as the reference standard (Supplemental Table 3). Inter-reader agreement between the two experts was perfect (κ = 1.00), while agreement between the AI model and expert consensus was substantial (κ = 0.71, 95% CI: 0.18–1.00). The AI model achieved a sensitivity of 96.2% (95% CI: 80.4–99.9%) and a specificity of 75.0% (95% CI: 19.4–99.4%) for identification of layered plaque presence. Representative examples of layered plaque segmentation are shown in Fig. 2, Supplemental Fig. 1 and Supplemental Video 1.

**Fig. 2:**
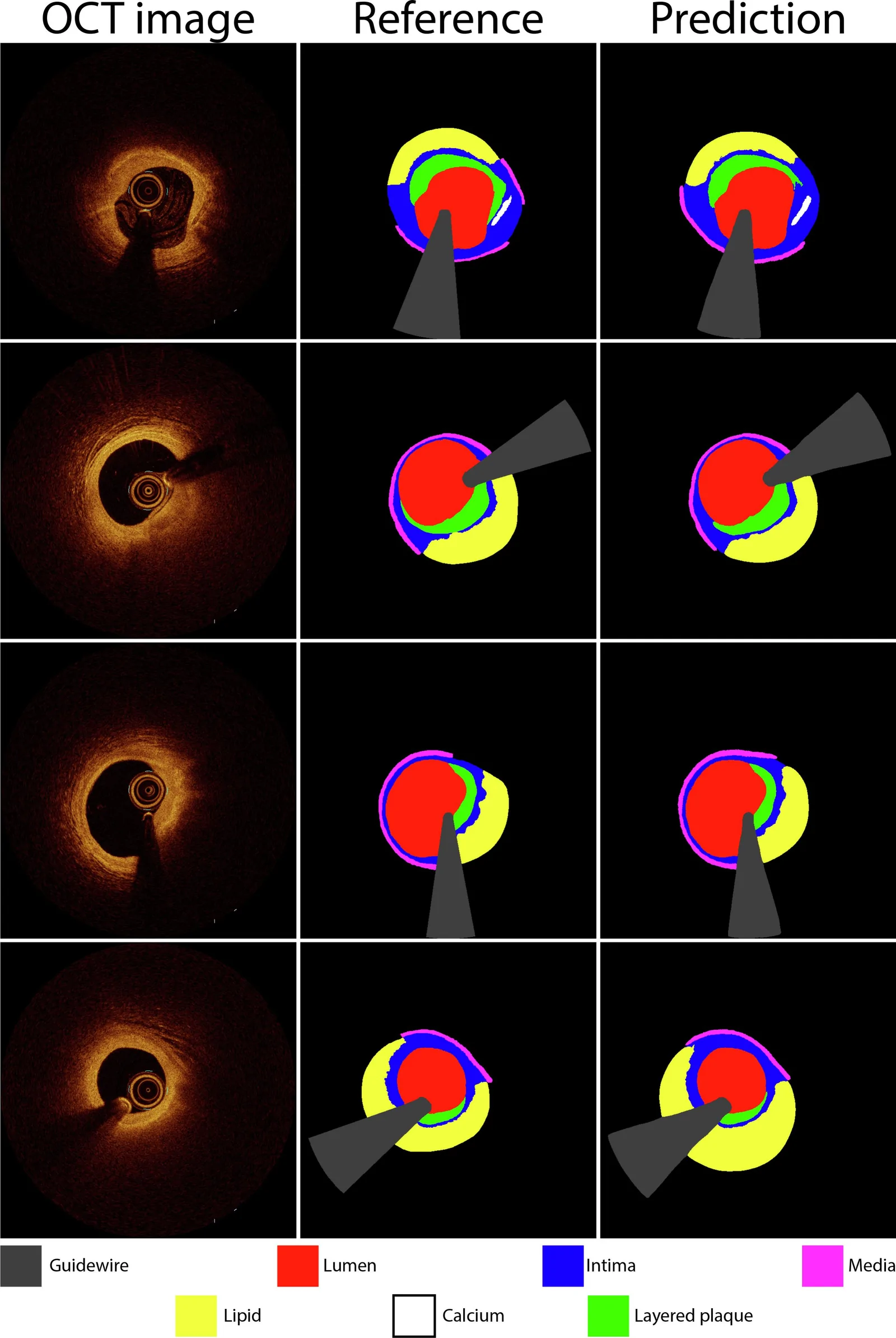
Representative segmentation predictions of layered plaque. Multiclass semantic segmentation examples, with an optical coherence tomography image (left column), reference standard (middle column) and prediction by OCT-AID (right column), with color legend shown at the bottom. OCT, optical coherence tomography.

### 3.3 Clinical descriptive baseline characteristics

The PECTUS-AI cohort included 414 patients with a mean age of 63.2 ± 10.3 years, of whom 79 patients (19.1%) were women. Baseline lipid-lowering therapy was used by 108 patients (26.1%). Clinical presentation included 213 patients with ST-elevation myocardial infarction (STEMI) (51.4%) and 201 with non-ST-elevation myocardial infarction (NSTEMI) (48.6%). Hypertension was present in 219 patients (52.9%) and diabetes in 60 (14.5%). All baseline characteristics are shown in Table 2.

**Table 2:** Layered plaque baseline characteristics patient level.

Variable	All patients (*n* = 414)	Non-layered plaque patients (*n* = 48)	Layered plaque patients (*n* = 366)	*P*-value
Age, years	63.2 ± 10.3	66.0 ± 8.8	62.8 ± 10.4	**0.020**
Female sex	79 (19.1%)	8 (16.7%)	71 (19.4%)	0.651
Hypertension	219 (52.9%)	22 (45.8%)	197 (53.8%)	0.297
Diabetes	60 (14.5%)	5 (10.4%)	55 (15.0%)	0.394
Prior myocardial infarction	63 (15.2%)	6 (12.5%)	57 (15.6%)	0.577
Prior stroke	8 (1.9%)	3 (6.3%)	5 (1.4%)	**0.021**
Baseline LLT	108 (26.1%)	13 (27.1%)	95 (26.0%)	0.867
*Clinical presentation*				**0.018**
STEMI NSTEMI	213 (51.4%) 201 (48.6%)	17 (35.4%) 31 (64.6%)	196 (53.6%) 170 (46.4%)	

Baseline patient characteristics stratified by layered plaque presence, with *p*-values for between-group comparisons.

*LLT, lipid-lowering therapy; NSTEMI, non-ST-segment elevation myocardial infarction; STEMI, ST-segment elevation myocardial infarction.*

Overall, layered plaques were identified in 366 patients (88.4%). Patients with layered plaques were slightly younger than those without (62.8 ± 10.4 vs. 66.0 ± 8.8 years; *p* *=* *0.020*). The distribution of female sex, hypertension, diabetes mellitus, prior myocardial infarction, and baseline lipid-lowering therapy was similar between groups (all *p* *>* *0.05*), whereas prior stroke was more common in patients without layered plaques (4.7% vs. 1.4%; *p* *=* *0.027*). Patients with layered plaques more frequently presented with STEMI than NSTEMI (53.6% vs. 46.4%), and within the STEMI subgroup layered plaques were more common than non-layered plaques (53.6% vs. 35.4%; *p* *=* *0.018*). Among patients with layered plaque, further stratification by the number of layered plaques showed that 52 patients (12.6%) had one layered plaque, whereas 314 patients (75.9%) had two or more layered plaques.

### 3.4 Clinical descriptive layered plaque analysis

Across 488 non-culprit lesions from the 414 patients, layered plaques were highly prevalent, identified in 424 non-culprit lesions (86.8%) (Supplemental Table 4). The median volume of a single layered plaque was 1.04 mm^3^ (IQR 0.42–2.39), with an average maximum layered plaque angle of 138 ± 62° and a maximum thickness of 621 ± 258 μm. Total layered plaque volume per lesion was 1.90 mm^3^ (IQR 0.67–4.21).

Layered plaque lesions demonstrated more advanced atherosclerotic characteristics compared with non-layered plaque lesions (Table 3). Lipid-rich plaque was more frequently observed in lesions with layered plaques (76.4% vs. 60.9%; *p* *=* *0.008*). Similarly, TCFA prevalence was higher in layered plaque lesions compared with non-layered lesions (29.2% vs. 15.6%; *p* *=* *0.016*), and macrophage accumulation was more common (25.0% vs. 9.4%; *p* *=* *0.010*). Cholesterol clefts were also exclusively observed in the layered plaque group (0.0% vs. 8.3%; *p* *=* *0.009*). After adjustment for age, prior stroke and clinical presentation using multivariable GEE models, the associations between layered plaque and lipid-rich plaque (*p* *=* *0.038*), TCFA (*p* *=* *0.028*), and macrophage accumulation (*p* *=* *0.027*) remained statistically significant.

**Table 3:** Layered plaque descriptive analysis.

	Non-layered plaque lesions (*n* = 64)	Layered plaque lesions (*n* = 424)	Unadjusted *p*-value	Adjusted p-value
Angle (°)	–	138 ± 62		
Thickness (μm)	–	621 ± 258		
Length (mm)	–	2.44 ± 2.00		
Maximum volume (mm^3^)	–	1.04 (IQR: 0.42–2.39)		
Total volume (mm^3^)	–	1.90 (IQR: 0.67–4.21)		
Lipid-rich plaque	39 (60.9%)	324 (76.4%)	**0.008**	**0.038**
TCFA	10 (15.6%)	124 (29.2%)	**0.016**	**0.028**
Macrophage accumulation	6 (9.4%)	106 (25.0%)	**0.010**	**0.027**
Thrombus	4 (6.3%)	39 (9.2%)	0.44	0.57
Neovascularization	7 (10.9%)	71 (16.7%)	0.25	0.22
Cholesterol clefts	0 (0.0%)	35 (8.3%)	**0.009**^a^	N/A^a^
Plaque rupture	2 (3.1%)	46 (10.8%)	0.080	0.075

Lesion-level layered plaque measurements and OCT vulnerability features stratified by layered plaque presence, with *p*-values for between-group comparisons. Adjusted *p*-values were obtained from multivariable GEE models adjusted for age, prior stroke, and clinical presentation, accounting for within-patient lesion clustering.

TCFA, thin-cap fibroatheroma.

a Adjusted GEE not applicable due to absence in non-layered group; Unadjusted GEE was performed using Fisher's exact test.

When stratifying lesions into three groups (non-layered plaques, lesions with one layered plaque, and lesions with ≥2 plaques), 69 lesions (14.1%) contained one layered plaque and 355 lesions (72.8%) contained two or more layered plaques. Morphologically, lesions with ≥2 layered plaques exhibited more extensive layered plaque burden than those with a single layered plaque, including larger maximum layered plaque angle (143 ± 62° vs. 114 ± 56°; *p* *<* *0.001*), greater layered plaque thickness (645 ± 256 μm vs. 500 ± 234 μm; *p* *<* *0.001*), longer layered plaque length (2.56 ± 1.91 mm vs. 1.81 ± 2.31 mm; *p* *=* *0.014*), and larger single layered plaque volume (1.98 ± 2.33 mm^3^ vs. 1.25 ± 2.37 mm^3^; *p* *=* *0.024*).

A similar pattern was observed for vulnerability features in the three-group comparison, with significant differences for lipid-rich plaque (*p* *=* *0.006*), TCFA (*p* *=* *0.006*), macrophage accumulation (*p* *=* *0.003*), and cholesterol clefts (*p* *=* *0.011*). No significant differences were observed for thrombus, neovascularization, or plaque rupture in either the two-group or three-group analyses. All OCT-derived characteristics for the three groups are summarized in Fig. 3.

**Fig. 3:**
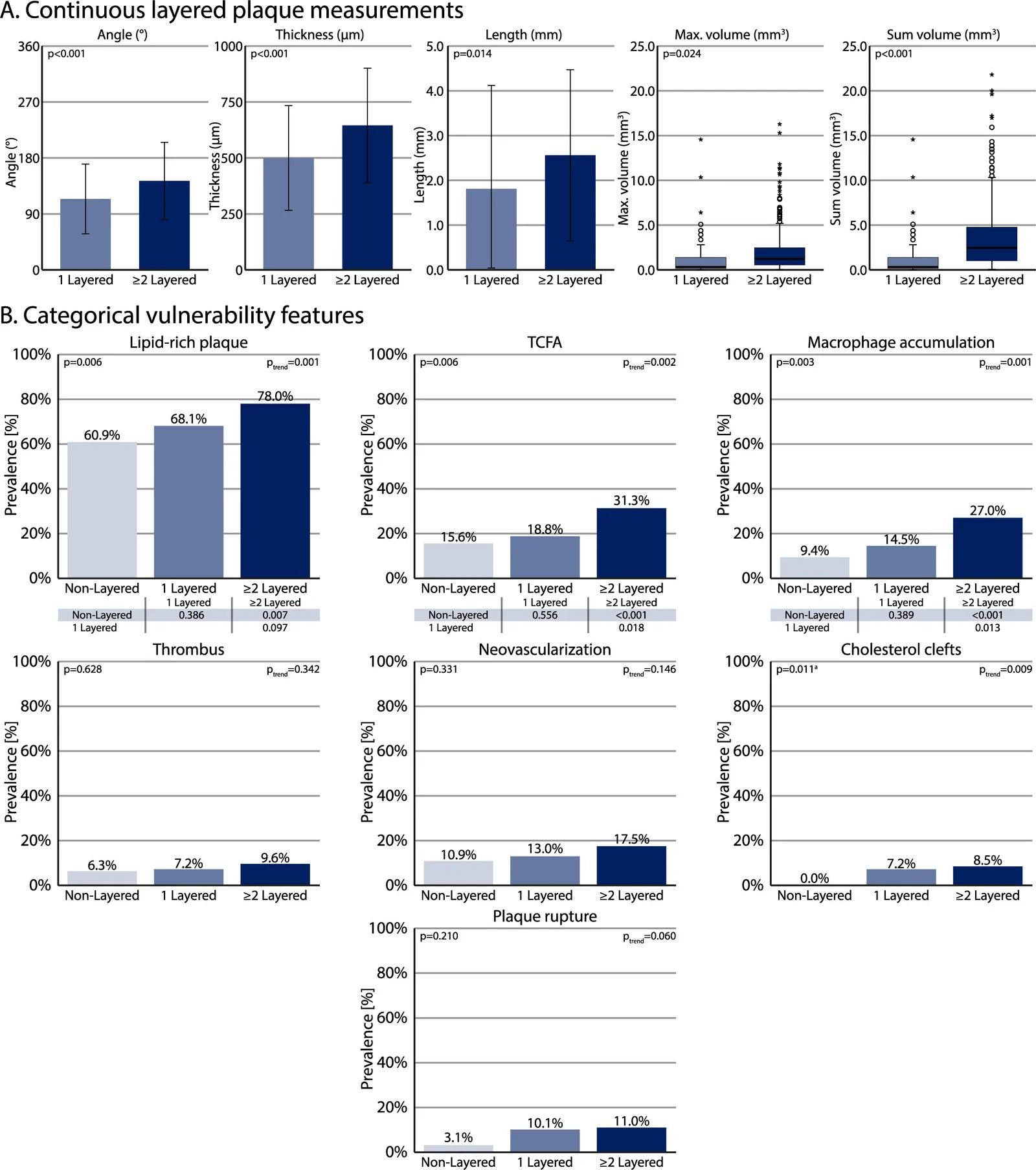
Association between layered plaque burden and OCT-derived vulnerability features. ^a^ GEE not applicable due to absence in non-layered group; Fisher–Freeman–Halton exact test applied. Stratification of lesions into non-layered plaque (64 lesions), single layered plaque (69 lesions), or ≥ 2 layered plaques (355 lesions). (A) Continuous layered plaque characteristics (angle, thickness, and length) are reported as mean ± SD. Maximum layered plaque volume and total layered plaque volume per lesion are reported as median (IQR). (B) OCT-derived vulnerability features are shown as prevalence (%) across lesions stratified by layered plaque burden (none, single, or ≥ 2 layered plaques). GEE, generalized estimating equation; OCT, optical coherence tomography; TCFA, thin-cap fibroatheroma.

## 4 Discussion

In this study, we extended a previously validated OCT-based AI segmentation model to include layered plaque as a dedicated class within a multiclass framework. Using this approach, we demonstrate that fully automated 3D segmentation and quantification of layered plaque is feasible. Applied to non-culprit lesions in patients with recent myocardial infarction, layered plaque was found to be highly prevalent and strongly associated with features of plaque vulnerability, including lipid-rich plaque, TCFA, and macrophage accumulation, suggesting an advanced atherosclerotic phenotype. These findings provide the first comprehensive in vivo volumetric and morphological characterization of layered plaques, establishing a framework for future studies.

Previous studies have largely focused on culprit lesions or relied on limited manual assessment of selected non-culprit segments. [9] Araki et al. manually examined layered plaque at both culprit and non-culprit sites and identified stable angina as a strong independent predictor at each location. [21] Yin et al. investigated layered plaque in non-culprit lesions within a small, predominantly NSTEMI cohort (89.4%), and observed strong associations with lipid-rich plaque and macrophage accumulation. [22] In our larger dataset of patients after myocardial infarction, we observed similar patterns, supporting the relation between layered plaque and established markers of vulnerability. Notably, by applying our AI-based analysis, we identified a high prevalence of layered plaques, exceeding what has been reported in manually assessed cohorts.

The biological interpretation of layered plaque supports our observations. Many plaque ruptures occur silently, triggering a healing response characterized by thrombus organization and collagen remodeling. Histopathologic studies show that this process results in distinct layers of dense collagen interspersed with smooth-muscle cells and proteoglycans, consistent with the layered appearance on OCT. [8] A serial OCT study of non-culprit lesions showed that plaques developing new layered patterns exhibit greater luminal narrowing, increased fibrous cap thickness, and reduced lipid content over time compared with plaques without layering. Importantly, patients with new layered patterns experience higher long-term rates of non-culprit-related major adverse cardiac events, primarily driven by clinically indicated revascularization. These findings underscore the clinical relevance of identifying layered plaque as a marker of prior subclinical rupture and ongoing plaque evolution. [23] However, layered plaque may also occur after subclinical plaque erosion. We postulate that layered plaques with adjacent lipid-rich plaque may represent prior plaque rupture, while layered plaques in absence of adjacent lipid may represent healing following plaque erosion. This hypothesis requires further confirmation using serial imaging.

Cholesterol clefts were observed exclusively in lesions containing layered plaque, further supporting the concept that layered plaque reflects advanced and complex atherosclerotic remodeling and repeated cycles of plaque injury and healing. Given the low absolute prevalence of cholesterol clefts, this observation should be considered hypothesis-generating and warrants confirmation in larger cohorts.

Despite its importance, assessment of layered plaque currently relies on expert visual interpretation, which introduces variability and risk of misclassification. In our dataset, inter-observer agreement for frame-level identification of layered plaque was substantial (κ = 0.79, 95% CI: 0.78–0.81), indicating that some degree of subjectivity remains even among expert readers. At the pullback level, inter-observer agreement was perfect (κ = 1.00), suggesting that observer variability may have less impact on the identification of layered plaque presence at the pullback level than on frame-level assessment. Until now, only a few AI-based analyses of layered plaques have been published. Araki et al. proposed a frame-level classification approach that demonstrated high sensitivity, but lacked the spatial resolution and volumetric capabilities of pixel-level segmentation. [11] The only previously published segmentation model, by Shibutani et al., reported modest performance (IoU 0.36) and was limited by a single-class design, a small dataset, and absence of clinically relevant analyses. [12] In contrast, our multiclass segmentation model not only improves performance, but also enables extraction of detailed morphological and volumetric features for each component and links layered plaque to established vulnerability markers. As layered plaque is a frequently encountered phenotype, its explicit modeling likely reduced misclassification onto adjacent classes, which may have contributed to the improved performance observed compared to the previously published segmentation model. [15]

By performing volumetric analysis across all frames within a lesion, our approach overcomes the observer bias inherent in manual frame selection and provides a more complete and reproducible characterization of layered plaque. This automated framework enables large-scale, unbiased studies of plaque healing and vulnerability and supports the concept of layered plaque as a marker of advanced plaque remodeling that warrants further investigation as a potential high-risk phenotype. However, OCT-derived vulnerability features do not necessarily reflect physiological lesion significance. [24] Future studies integrating automated plaque characterization with physiological assessment and clinical outcomes are needed to determine the clinical relevance of layered plaque.

### 4.1 Limitations

This study has a few limitations. First, although frame-level specificity was high, frame-level sensitivity was modest, which may have resulted in underdetection of layered plaque at the frame-level. As a result, the prevalence and extent of layered plaque may have been underestimated. Furthermore, underdetection of a subset of layered plaques may have influenced the descriptive analyses. Second, histology-based validation rather than OCT expert consensus would be the ideal reference standard for model training. To mitigate the limitation of expert interobserver variability, we used a multi-reader approach to reduce the risk of misclassification. Third, although the model was evaluated using separate training, validation, and test datasets, all data originated from the ORANGE and PECTUS-obs studies. External validation in independent cohorts acquired at different centers is required to confirm generalizability before clinical implementation. Fourth, the ground-truth annotations for model development and validation were generated by trained analysts under expert supervision rather than through independent multi-reader pixel-level annotation. As a result, interobserver agreement for pixel-level layered plaque segmentation was not formally assessed.

## 5 Conclusion

In this study, we expanded our OCT-AID model to enable robust pixel-level segmentation of layered plaque. Using this approach, we characterized layered plaques in non-culprit lesions of patients with recent myocardial infarction, showing higher prevalence of lipid-rich plaque, TCFA, and macrophage accumulation in patients with layered plaque. These findings show that layered plaque is closely associated with established markers of plaque vulnerability, supporting further investigation of its potential prognostic relevance in patients with coronary artery disease.

## CRediT authorship contribution statement

**Ruben G.A. van der Waerden:** Data curation, Formal analysis, Investigation, Methodology, Software, Visualization, Writing – original draft. **Joske L. van der Zande:** Data curation, Writing – review & editing. **Simone Saitta:** Supervision, Writing – review & editing. **Rick H.J.A. Volleberg:** Data curation, Writing – review & editing. **Jan-Quinten Mol:** Data curation, Writing – review & editing. **Pierandrea Cancian:** Software, Writing – review & editing. **Thijs J. Luttikholt:** Writing – review & editing. **Xiaojin Gu:** Writing – review & editing. **Leah Heil:** Writing – review & editing. **Maarten van Leeuwen:** Data curation, Writing – review & editing. **Martijn Meuwissen:** Data curation, Writing – review & editing. **Alexey V. Protopopov:** Data curation, Writing – review & editing. **Peep Laanmets:** Data curation, Writing – review & editing. **Oleg V. Krestyaninov:** Data curation, Writing – review & editing. **Robert Dennert:** Data curation, Writing – review & editing. **Rohit M. Oemrawsingh:** Data curation, Writing – review & editing. **Jan-Peter van Kuijk:** Data curation, Writing – review & editing. **Karin Arkenbout:** Data curation, Writing – review & editing. **Dirk J. van der Heijden:** Data curation, Writing – review & editing. **Elvin Kedhi:** Data curation, Writing – review & editing. **Tomasz Roleder:** Data curation, Writing – review & editing. **Clara I. Sánchez:** Writing – review & editing. **Bram van Ginneken:** Writing – review & editing. **Ivana Išgum:** Funding acquisition, Supervision, Writing – review & editing. **Jos Thannhauser:** Supervision, Writing – review & editing. **Niels van Royen:** Funding acquisition, Supervision, Writing – review & editing.

## Funding

This publication is part of the project ROBUST: Trustworthy AI-based Systems for Sustainable Growth with project number KICH3.LTP.20.006, which is (partly) financed by the Dutch Research Council (NWO), Abbott, and the Dutch Ministry of Economic Affairs and Climate Policy (EZK) under the program LTP KIC 2020-2023. Funding partners were not involved in the collection, analysis and interpretation of the data, and in the preparation, review, or approval of the manuscript.

All content represents the opinion of the authors, which is not necessarily shared or endorsed by their respective employers and/or sponsors.

PECTUS-obs was financially supported by Abbott and Health∼Holland (grant number LSHM19102). Funding parties were not involved in the design and conduct of the study; collection, management, analysis, and interpretation of the data; preparation, review and approval of the manuscript; and decision to submit the manuscript for publication.

## Declaration of competing interest

Dr. Sanchez reports institutional grants from Abbott. Dr. van Ginneken is shareholder from Thirona and Plain Medical. Dr. Išgum reports institutional grants from the Dutch Science Foundation, Abbott, Pie Medical Imaging BV, Philips, and Esaote SpA. Dr. van Royen reports institutional research grants from Abbott, Philips, Biotronik, and Medtronic.

All other authors have reported that they have no relationships relevant to the contents of this paper to disclose.

## Data Availability

The datasets used and/or analyzed during the current study are available from the corresponding author on reasonable request. The following are the supplementary data related to this article.Supplementary Video 1Multiclass semantic segmentation of layered plaques in an optical coherence tomography pullback. The left panel shows the original OCT pullback video, while the right panel displays the corresponding OCT-AID predictions overlaid on the video frames.Supplementary material Multiclass semantic segmentation of layered plaques in an optical coherence tomography pullback. The left panel shows the original OCT pullback video, while the right panel displays the corresponding OCT-AID predictions overlaid on the video frames. Supplementary material Supplementary data to this article can be found online at https://doi.org/10.1016/j.ijcha.2026.102025.
