# Supplementary material for: Automated segmentation and clinical characterization of layered plaque in intracoronary optical coherence tomography
Source: Int J Cardiol Heart Vasc. 2026 Oct 9;67:102025. doi: 10.1016/j.ijcha.2026.102025 (PMC13677019; doi:10.1016/j.ijcha.2026.102025)
Supplement: Supplementary file 1 [file mmc2.docx]

Supplemental material

Content

[Supplemental Figures and Videos	3](#_Toc239921118)

[Supplemental Figure 1	3](#_Toc239921119)

[Supplemental Video 1	4](#_Toc239921120)

[Supplemental Tables	5](#_Toc239921121)

[Supplemental Table 1	5](#_Toc239921122)

[Supplemental Table 2	6](#_Toc239921123)

[Supplemental Table 3	7](#_Toc239921124)

[Supplemental Table 4	8](#_Toc239921125)

## Supplemental Figures and Videos

### Supplemental Figure 1

Multiclass semantic segmentation examples illustrating missed or incomplete predictions of layered plaques. Each row shows the optical coherence tomography image (left column), reference standard (middle column) and prediction by OCT-AID (right column), with color legend shown at the bottom.

### Supplemental Video 1

## Supplemental Tables

### Supplemental Table 1

| **Hyperparameter** | **Value** |
| --- | --- |
| Model architecture | U-net |
| Input image coordinate | Cartesian |
| Input image shape | 704 x 704 x 7 x 1 |
| Output image shape | 704 x 704 x 1 |
| Batch size | 5 |
| Initial learning rate | 0.01 |
| Learning rate scheduler | Polynomial |
| Optimizer | Stochastic gradient descent |
| Epochs | 1000 |
| Loss function | Equally weighted Dice and Cross-Entropy, including deep supervision |
| Filter size | [32, 64, 128, 256, 512, 512, 512, 512] |
| Folds | 5 |

Model architecture and training hyperparameters for the OCT‑AID layered plaque segmentation model.

### Supplemental Table 2

| **Variable** | **Training set (n=241)** | **Held-out test set (n=26)** | **p-value** |
| --- | --- | --- | --- |
| Age, years | 63.69±9.87 | 65.41±9.98 | 0.860 |
| Female sex | 50 (20.7%) | 8 (30.8%) | 0.239 |
| Hypertension | 108 (44.8%) | 11 (42.3%) | 0.807 |
| Diabetes | 38 (15.8%) | 3 (11.5%) | 0.570 |
| Prior myocardial infarction | 36 (14.9%) | 6 (23.1%) | 0.279 |
| Prior stroke | 7 (2.9%) | 1 (3.8%) | 0.789 |
| Baseline LLT | 76 (31.5%) | 7 (26.9%) | 0.629 |
| *Clinical presentation* |  |  | **0.033** |
| STEMI | 103 (42.7%) | 11 (42.3%) |  |
| NSTEMI | 127 (52.9%) | 14 (53.8%) |  |
| CCS | 8 (3.3%) | 0 (0.0%) |  |
| UAP | 3 (1.3%) | 0 (0.0%) |  |
| Cardiac Arrest | 0 (0.0%) | 1 (3.8%) |  |
| *Dataset origin* |  |  | 0.906 |
| PECTUS-obs | 206 (85.5%) | 22 (84.6%) |  |
| ORANGE | 35 (14.5%) | 4 (15.4%) |  |

Baseline patient characteristics of the model development cohort stratified by training and test set, with p‑values for between‑group comparisons.

*CCS, chronic coronary syndrome; UAP, unstable angina pectoris; LLT, lipid-lowering therapy; NSTEMI, non-ST-segment elevation myocardial infarction; STEMI, ST-segment elevation myocardial infarction.*

### Supplemental Table 3

| **Layered plaque presence** | **Kappa (95% CI)** | **Valid frames/pullbacks** | **Layered plaque present (n; %)** | **False negative (n; %)** | **False positive (n; %)** | **Sensitivity (%; 95% CI)** | **Specificity (%; 95% CI)** |
| --- | --- | --- | --- | --- | --- | --- | --- |
| *Frame-level* |  |  |  |  |  |  |  |
| Inter-reader agreement (observer 1 reference) | 0.79 (0.78-0.81) | 11,143 | 1,919 (17.2%) | 305 (3.3%) | 355 (3.8%) | 84.1 (82.4-85.7) | 96.2 (95.7-96.5) |
| Inter-reader agreement (observer 2 reference) | 0.79 (0.78-0.81) | 11,143 | 1,969 (17.7%) | 355 (3.8%) | 305 (3.3%) | 82.0 (80.2–83.6) | 96.7 (96.3–97.0) |
| AI vs. consensus | 0.52 (0.50–0.55) | 10,483 | 1614 (15.4%) | 843 (8.0%) | 267 (2.6%) | 47.8 (45.3–50.2) | 97.0 (96.6–97.3) |
| *Pullback-level* |  |  |  |  |  |  |  |
| Inter-reader agreement | 1.00 | 30 | 26 (86.7%) | 0 (0.0%) | 0 (0.0%) | **-** | **-** |
| AI vs. consensus | 0.71 (0.18-1.00) | 30 | 26 (86.7%) | 1 (3.8%) | 1 (3.8%) | 96.2 (80.4-99.9) | 75.0 (19.4-99.4) |

Inter-reader agreement was assessed between two independent readers who evaluated each pullback in the held-out test set for the presence or absence of layered plaque. AI performance was assessed against the consensus of both readers, which served as the reference standard. Consensus layered plaque present refers to frames/pullbacks classified as containing layered plaque by both readers in the pullback.

*AI, artificial intelligence; CI, confidence interval*

### Supplemental Table 4

| **Variable** | **All lesions (n=488)** | **Non-layered plaque lesions (n=64)** | **Layered plaque lesions (n=424)** | **P-value** |
| --- | --- | --- | --- | --- |
| Age, years | 63.26±10.32 | 66.11±9.01 | 62.83±10.44 | **0.040** |
| Female sex | 88 (18.0%) | 8 (12.5%) | 80 (18.9%) | 0.297 |
| Hypertension | 256 (52.5%) | 28 (43.8%) | 228 (53.8%) | 0.290 |
| Diabetes | 79 (16.2%) | 8 (12.5%) | 71 (16.7%) | 0.690 |
| Prior myocardial infarction | 76 (15.6%) | 8 (12.5%) | 68 (16.0%) | 0.409 |
| Prior stroke | 9 (1.8%) | 3 (4.7%) | 6 (1.4%) | **0.027** |
| Baseline LLT | 130 (26.6%) | 18 (28.1%) | 112 (26.4%) | 0.842 |
| *Clinical presentation* |  |  |  | **0.032** |
| STEMI  NSTEMI | 248 (50.8%)  240 (49.2%) | 21 (32.8%)  43 (67.2%) | 227 (53.5%)  197 (46.5%) |  |

Baseline lesion characteristics stratified by layered plaque presence, with p‑values for between‑group comparisons.

*LLT, lipid-lowering therapy; NSTEMI, non-ST-segment elevation myocardial infarction; STEMI, ST-segment elevation myocardial infarction.*
